# Atorvastatin protects against contrast-induced acute kidney injury via upregulation of endogenous hydrogen sulfide

**DOI:** 10.1080/0886022X.2020.1740098

**Published:** 2020-03-18

**Authors:** Lin Yan, Lin Jiaqiong, Guo Yue, Li Xiaoyong, Tan Xuexian, Long Ming, Li Yinglan, Liao Xinxue, Huang Zena

**Affiliations:** a Department of Nephrology, Third Affiliated Hospital, Guangzhou Medical University, Guangzhou, China; b Department of Medical Genetics, School of Basic Medical Sciences, Southern Medical University, Guangzhou, China; c Department of Cardiology, First Affiliated Hospital, Sun Yat-sen University, Guangzhou, China; d Department of General Surgery, Third Affiliated Hospital, Guangzhou Medical University, Guangzhou, China; e Department of Pathology, Third Affiliated Hospital, Guangzhou Medical University, Guangzhou, China; g Department of Endocrine, Guangdong General Hospital, Guangdong Academy of Medical Sciences, Guangzhou, China; i Department of General Medicine, Guangdong General Hospital, Guangdong Academy of Medical Sciences, Guangzhou, China

**Keywords:** Contrast medium, acute kidney injury, atorvastatin, hydrogen sulfide

## Abstract

**Background:**

Contrast-induced acute kidney injury (CIAKI) is the third leading cause of acute renal failure in hospitalized patients. This study was aimed to investigate whether atorvastatin could upregulate the expression of hydrogen sulfide (H_2_S) and hence protect against CIAKI.

**Methods:**

We treated male rats and NRK-52E cells by iopromide to establish *in vivo* and *in vitro* models of CIAKI. Pretreatment with atorvastatin was given in CIAKI rats to investigate its effect on CIAKI. We collected serum and urine samples to detect renal function. We obtained kidney tissue for histological analysis and detection of protein concentration. We tested the serum concentration of H_2_S and renal expression of two H_2_S synthetases [cystathionine γ-lyase (CSE) and cystathionine-β synthase (CBS)]. NaHS was pretreated in NRK-52E cells to testify its underlying effect on contrast-induced injury.

**Results:**

Atorvastatin significantly ameliorated renal dysfunction and morphological changes in CIAKI rats, as well as inflammation, apoptosis, and excessive oxidative stress. Atorvastatin also markedly increased the serum concentration of H_2_S and renal expression of CSE and CBS. Moreover, pretreatment with NaHS in NRK-52E cells considerably attenuated contrast-induced cell death and inflammation.

**Conclusion:**

Atorvastatin protects against CIAKI *via* upregulation of endogenous hydrogen sulfide.

## Background

Contrast-induced acute kidney injury (CIAKI) is an important syndrome of acute renal failure occurring after the intravascular administration of iodinated contrast media (CM) in diagnostic and interventional procedures. It is defined as an increase in serum creatinine (SCr) level by more than 0.5 mg/dL or 25% above the baseline value within 72 h of CM injection [1]. CIAKI poses the third leading cause of acute renal failure in hospitalized patients, with an estimated 12% incidence [2]. About 10% of CIAKI patients need permanent dialysis, and many others will retain some degree of renal insufficiency [3]. Therefore, prevention of CIAKI shows predominant importance.

The β-hydroxy-β-methylglutaryl coenzyme A (HMG-CoA) inhibitors (statins) exert beneficial effects in different kidney diseases of animal models [4,5]. The effectiveness of statin pretreatment in reducing the incidence of CIAKI has been examined in some observational [6–8] and randomized studies [9–11]. According to recent studies [12,13], amelioration of oxidative stress, decreased synthesis of proinflammatory cytokines, inhibition of cell hypertrophy/proliferation, and enhanced expression of endogenous gasotransmitter may all contribute to the benefit of statin treatment in CIAKI.

Hydrogen sulfide (H_2_S), as a novel gasotransmitter, has been recognized as an important participant in modulating multiple organs [14], including renal function control [15]. It affects both vascular and tubular actions by increasing the glomerular filtration rate (GFR), urinary sodium and potassium excretion, and fractional excretion of sodium and potassium [16]. It can also mediate renal apoptosis [17]. In several studies, atorvastatin was demonstrated to increase the tissue expression of H_2_S, including kidney [18]. This prompted us to investigate whether statin could upregulate H_2_S expression and hence protect against CIAKI.

## Methods

### Animal studies

#### CIAKI in rats and pretreatment with atorvastatin

All animal experiments were approved by Guangzhou Medical University. CIAKI protocol was established based on previous studies [19,20], consisting of indomethacin (10 mg/kg, Sigma-Aldrich, USA), followed at 15 and 30 min, respectively, by N-nitro-L-arginine methyl ester (10 mg/kg, Sigma-Aldrich, USA) and iopromide (2.9 g/kg, Bayer Co., USA). Mature male Sprague–Dawley rats, weighting 180–200 g, were chosen for this experiment. They were housed under controlled conditions of light (12 h/12 h light/dark cycle) and temperature (21–23 °C) and allowed free access to standard laboratory diet and water. Rats were divided into four groups, while there are six rats in each group: (1) Control group: rats received saline solution at each time point; (2) CIAKI group: rats established CIAKI protocol; (3) CIAKI + Statin group: before establishment of CIAKI protocol, rats received atorvastatin (5 mg/kg, Pfizer Ltd., USA) intragastrically once daily for three consecutive days; (4) Statin group: rats only received atorvastatin treatment. The second day after CIAKI protocol establishment, rats were allocated into metabolic cages for an 24-h urine collection. After that, we weighted them and obtained blood samples. Finally, the rats were killed under diethyl ether anesthesia. The kidneys were weighted and bisected in the equatorial plane; the right kidney was divided for western blot analyses, and the left kidney was fixed in phosphate-buffered 4% formalin and prepared for routine histological examination.

#### Evaluation of biochemical parameter

Mean SCr, blood urea nitrogen (BUN), and urinary creatinine (UCr) concentrations were measured using automatic biochemistry analyzer at the Clinical Laboratory, The First Affiliated Hospital of Sun Yat-sen University. GFR was estimated from endogenous creatinine clearance (ClCr) using the standard formula: ClCr = UCr × urine volume/SCr. Creatinine clearance for 48 h after the injection of CM was calculated as ml/min.

ELISA kits were used to determine the concentrations of serum neutrophil gelatinase-associated lipocalin (NGAL, Beyotime Ltd., China), IL-1β (Telenbiotech, China), IL-18 (Telenbiotech, China), MCP-1 (Pepro Tech, USA), and H_2_S (Meimian Ltd., China). In order to estimate the systemic oxidative stress, serum MDA level was also tested by ELISA (Telenbiotech, China). Briefly, serum was centrifuged for 10 min at 3000 rpm. The supernatants were collected and 50 μL samples were added to the 96-well protein binding plate and incubated at 37 °C for 2 h. After washing two times with wash buffer, 100 μL of assay diluent per well was added and incubated for 2 h on an orbital shaker. Plates were then washed completely with thorough aspiration between each wash, 50 μL of the diluted antibodies were added to indicated wells, and incubated for 1 h. After washing three times with wash buffer, 50 μL of the diluted secondary antibody-HRP conjugate was added to each well and incubated for 1 h on an orbital shaker. Then 50 μL of substrate solution was added to each well and incubated for 15 min at room temperature on an orbital shaker. Finally, 50 μL of stop solution was added to terminate the reaction, and the absorbance of each well was recorded using a microplate reader at 450 nm.

#### Histological examination of renal tissues

Kidney sections of 4 μm were obtained from paraffin-embedded samples and subjected to hematoxylin and eosin (HE) staining and Periodic Acid-Schiff (PAS) staining. For semiquantitative analysis of morphological changes, 10 high-magnification (×200) fields of the cortex and outer stripe of the outer medulla were randomly selected. The abnormal tubular histopathology was calculated according to Weidemann’s study[21]: 0, no abnormalities; 1+, damage less than 25%; 2+, damage between 25 and 50%; 3+, damage between 50 and 75%; 4+, damage more than 75%. The average score of 10 fields was used for each kidney sample.

TUNEL assay was performed on paraffin-embedded kidney tissue to present apoptotic DNA fragmentation. Counterstained with DAPI, the apoptotic cells (FITC positive) appeared brightly green in color after fluorescein staining.

Immunohistochemical staining was performed in 4 μm kidney sections as described previously [22]. Primary antibodies used were active (cleaved) Caspase-3 as an index of apoptosis [23], as well as CSE and CBS presenting H_2_S generation. The areas stained for specific antibodies were quantified in 10 randomly selected fields (×200) per sample using the Image-Pro Plus Software (Media cybernetics, Bethesda, MD).

#### Western blot analysis

As previously described [24], nuclear and cytoplasmic proteins from kidney tissues were extracted, whose concentration was measured next. Equal amount of protein (40 μg) were separated by 12% SDS-PAGE and transferred to 0.45 μm polyvinylidene fluoride membranes. The membranes were blocked for 2 h with 5% skimmed milk at room temperature. Then, the membranes were incubated with primary antibodies specific to CSE(1:1000, Abcam Inc., USA), CBS (1:1000, Proteintech Inc., China), or GAPDH (1:10 000, Proteintech Inc., China) overnight at 4 °C. After washing three times, the membranes were probed with the secondary antibody (1:5000, Proteintech Inc., China) at room temperature for 1 h. Blots were then developed with the ECL Plus Western Blotting Detection System (Amersham Life Science, UK). Band intensities were quantified using Image J 1.47i software.

#### Measurement of renal MDA

Renal MDA level was measured according to a previous study [25]. Homogenates were centrifuged for 10 min at 1800 rpm. The supernatants were diluted 1:10 with PBS and homogenate was mixed with 3 mL 1%H_3_PO_4_ and 1 mL 0.672% TBA. After mixing, the solution was incubated for 1 h in a boiling water bath. Then the cooled tubes were centrifuged for 10 min at 1800 rpm. 200 μL supernatant was added into a 96-well plate and absorbance was read using a microplate reader at 532 nm (Molecular Devices, USA). Renal MDA levels were calculated as nmol MDA/g tissue.

### *In vitro* studies

#### Cell culture and treatments

NRK-52E cells (Jiniou Co., China) were maintained in Dulbecco’s modified Eagle’s medium (DMEM, Gibco, USA) and supplemented with 10% fetal bovine serum (Gibco, USA) in a 5% CO_2_ atmosphere at 37 °C. The culture medium was replaced with fresh medium every 2–3 days. The cells were expanded to new culture plates when about 80% confluent. In order to testify the effect of H_2_S on CM-induced injury, NRK-52E cells were pretreated with NaHS (Sigma-Aldrich, USA) of different concentrations (400–800 µM) for 0.5 h before exposure to CM.

#### Cell viability assay

The viability of cells was detected by CCK-8 assay. The NRK-52E cells were incubated in 96-well plate at a concentration of 1 × 10^4^ cells/ml at 37 °C. After the indicated treatments, the cells were washed twice with PBS. Then the cells were incubated with 10 µL CCK-8 test solution (Dojindo Lab., Japan) and 90 µL DMEM at 37 °C for 2 h. The optical density (OD) was measured by absorbance value at the 450 nm wavelength using a microplate reader (Molecular Devices, USA). The mean of the OD of three wells in each group was used for calculation of cellular activity percentage, according to the following formula: cell viability (%) = (OD treatment group/OD control group) × 100%.

#### Evaluation of IL-1β and IL-18 secretion

The NRK-52E cells were incubated in 96-well plates with indicated treatments. The levels of IL-1β and IL-18 in the culture supernatant were measured by ELISA.

**Figure 1.**
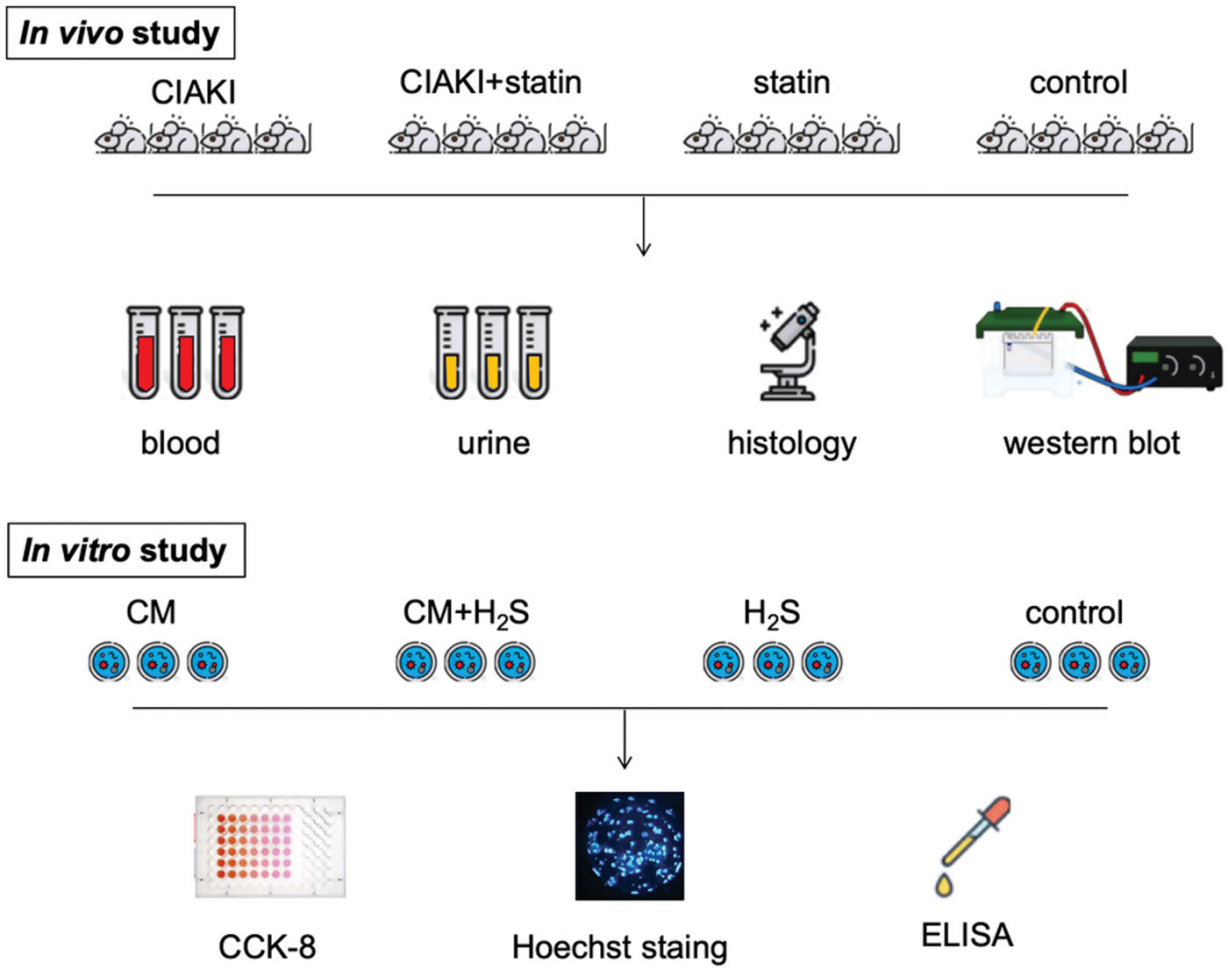
The whole study design for atorvastatin protecting against CIAKI *via* upregulation of H_2_S. The rats stand for Sprague–Dawley rats, and the cells stand for NRK-52E cells.

The whole study design is shown in Figure 1.

### Statistical analysis

In *in vivo* studies, there are six animals in each group. In *In vitro* studies, we ran each experiment triply. All data are expressed as the mean ± SD. Differences between groups are determined by one-way ANOVA using SPSS 21.0 software (SPSS, Inc, Chicago, IL, USA). *p* < 0.05 was considered significant throughout the analysis.

## Results

### Atorvastatin protects renal function in rat CIAKI model

**Figure 2.**
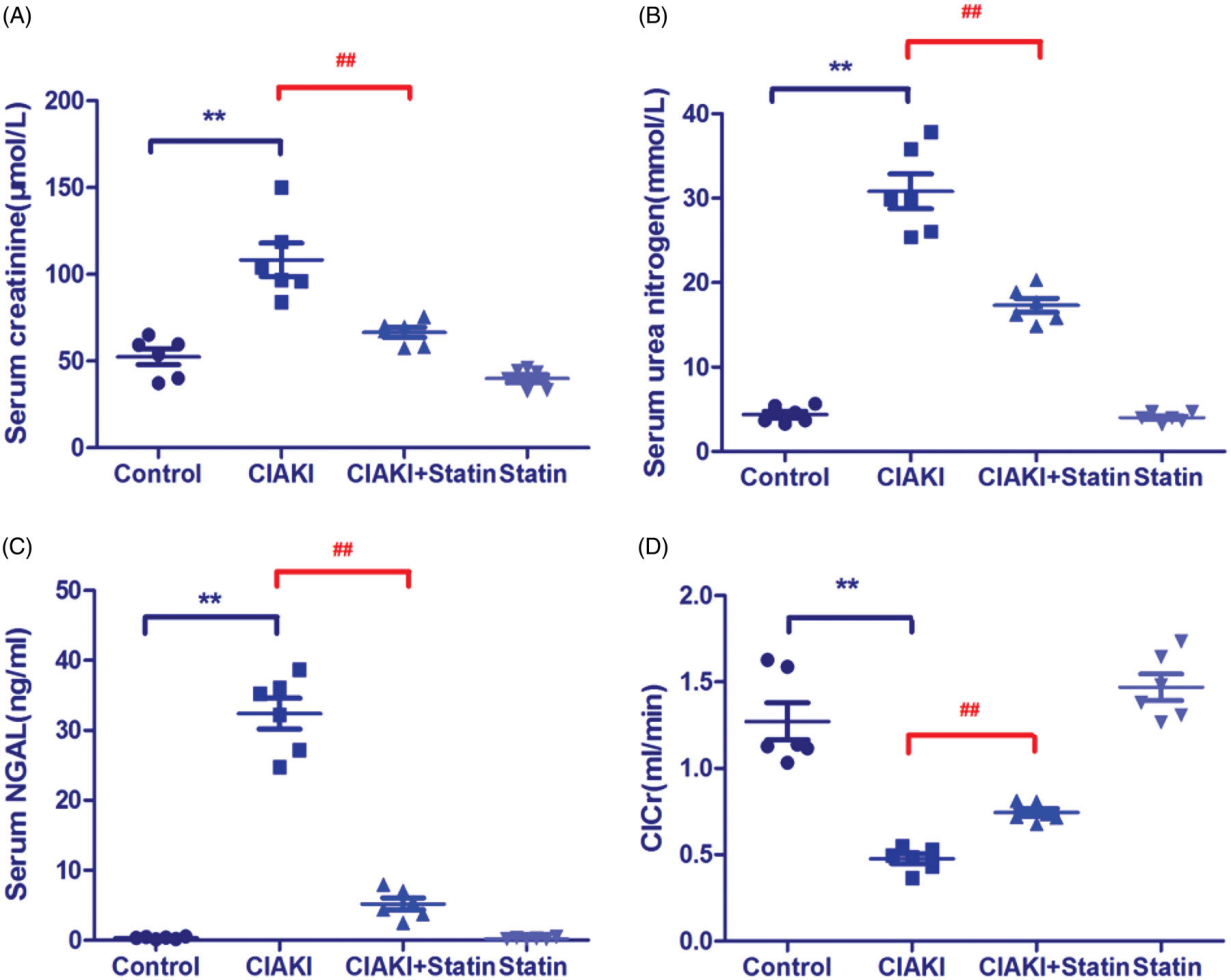
Atorvastatin protects renal function in rat CIAKI model. Rats were injected with iopromide to establish CIAKI model. 48 h after injection of iopromide, rats showed significant increase of SCr, BUN and serum NGAL, as well as decrease of ClCr. However, pretreatment with atorvastatin for 3d could alleviate the contrast-induced expression of SCr, BUN and serum NGAL, with restoring ClCr. Data are presented as mean ± SD (*n* = 6). ** *p* < 0.01 versus control group, ## *p* < 0.01 versus CIAKI group.

The rat CIAKI model was induced by administration of iopromide, based on inhibition of prostaglandin and nitric oxide synthesis. As shown in Figure 2, rats in CIAKI group manifested elevation in serum levels of Cr, BUN, and NGAL at 48 h after iopromide injection (*p* < 0.01), compared with the control group. Meanwhile, the ClCr decreased more than 50% after iopromide treatment (*p* < 0.01). However, pretreatment with atorvastatin considerably attenuated CM-induced deterioration of renal function, presenting a decrease of SCr, BUN, and NGAL levels (*p* < 0.01). Moreover, ClCr was also upregulated with atorvastatin treatment, compared with that of CIAKI group (*p* < 0.01). Atorvastatin alone had no effect on renal function.

### Atorvastatin protects renal morphology in rat CIAKI model

**Figure 3.**
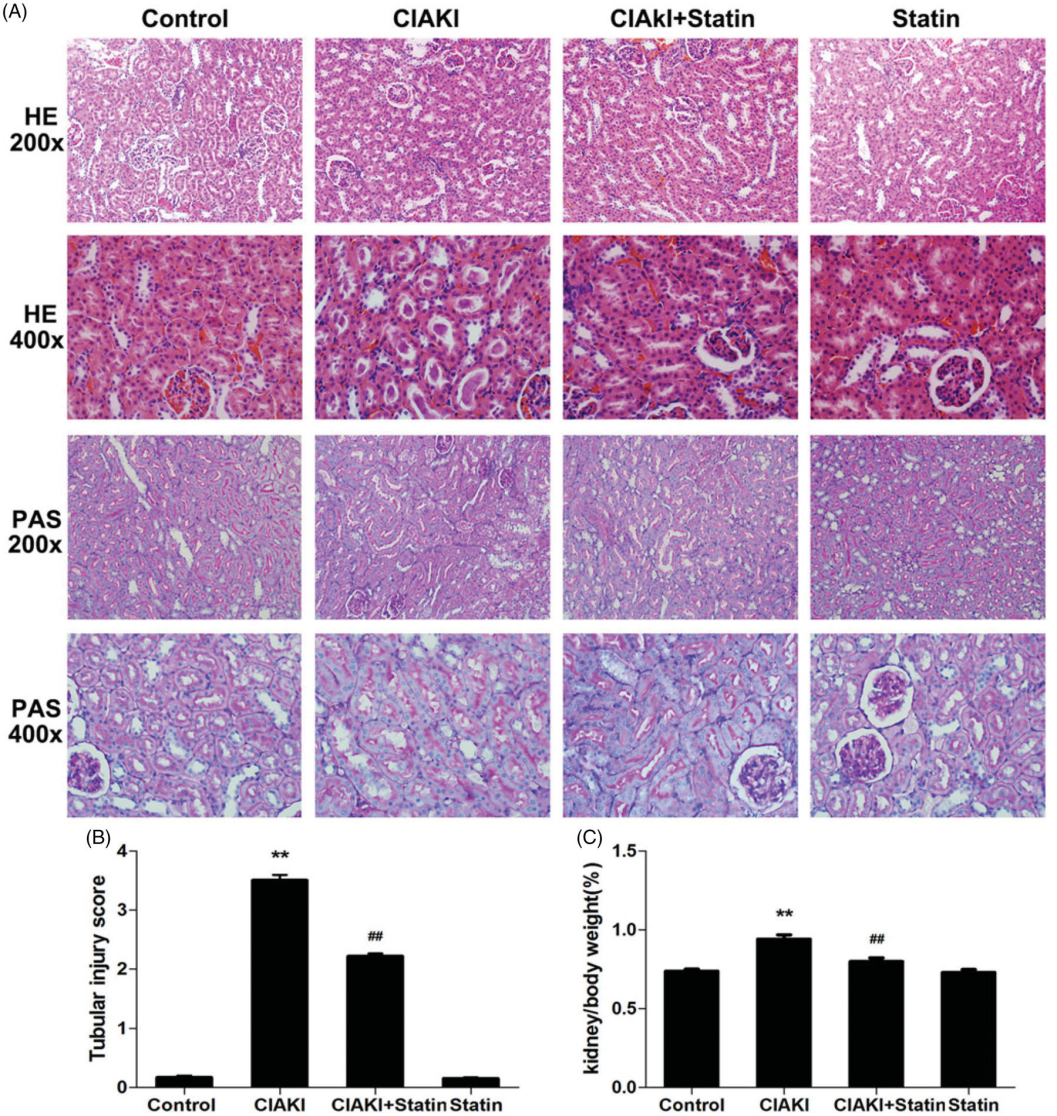
Atorvastatin protects renal morphology in rat CIAKI model. Representative photomicrographs of HE and PAS stained kidney sections (A), and semi-quantitative scoring analysis of tubular degeneration were presented (B). Marked tubular vacuolization and degeneration, necrosis, and hyaline or cellular casts associated with infiltration of mononuclear cells were shown. For the semi-quantitative analysis of morphological changes, 10 high magnification (×200) fields of the cortex and the outer stripe of the outer medulla in rats were randomly selected. The extent of tubular injury was then graded with a score from 0 to 4 points. Iopromide caused severe tubular injuries at 48 h after iopromide injection. However, pretreatment with atorvastatin remarkably attenuated the tubular injuries. The Kw/Bw was elevated at 48 h after iopromide injection (C), which could also be mitigated by atorvastatin. Data are presented as mean ± SD (*n* = 6). **p < 0.01 versus control group. ## *p* < 0.01 versus CIAKI group.

Next, we investigated the morphological change in CIAKI rat. As presented in Figure 3, the kidney weight to body weight ratio (Kw/Bw, Figure 3(C)) was increased in CIAKI group, which could be dampened by atorvastatin significantly, showing that atorvastatin could ameliorate CM-induced renal edema. Then, we studied about the histological alteration of rat kidney (Figure 3(A)). We found severe tubular necrosis, proteinaceous casts, interstitial edema, and medullary congestion in CM-treated renal section, by HE staining and PAS staining (Figure 3(A,B), *p* < 0.01). Nevertheless, rats with atorvastatin pretreatment showed markedly minor tubular and interstitial injuries (*p* < 0.01). Atorvastatin alone had no effect on renal morphology.

### Atorvastatin protects against apoptosis in rat CIAKI model

**Figure 4.**
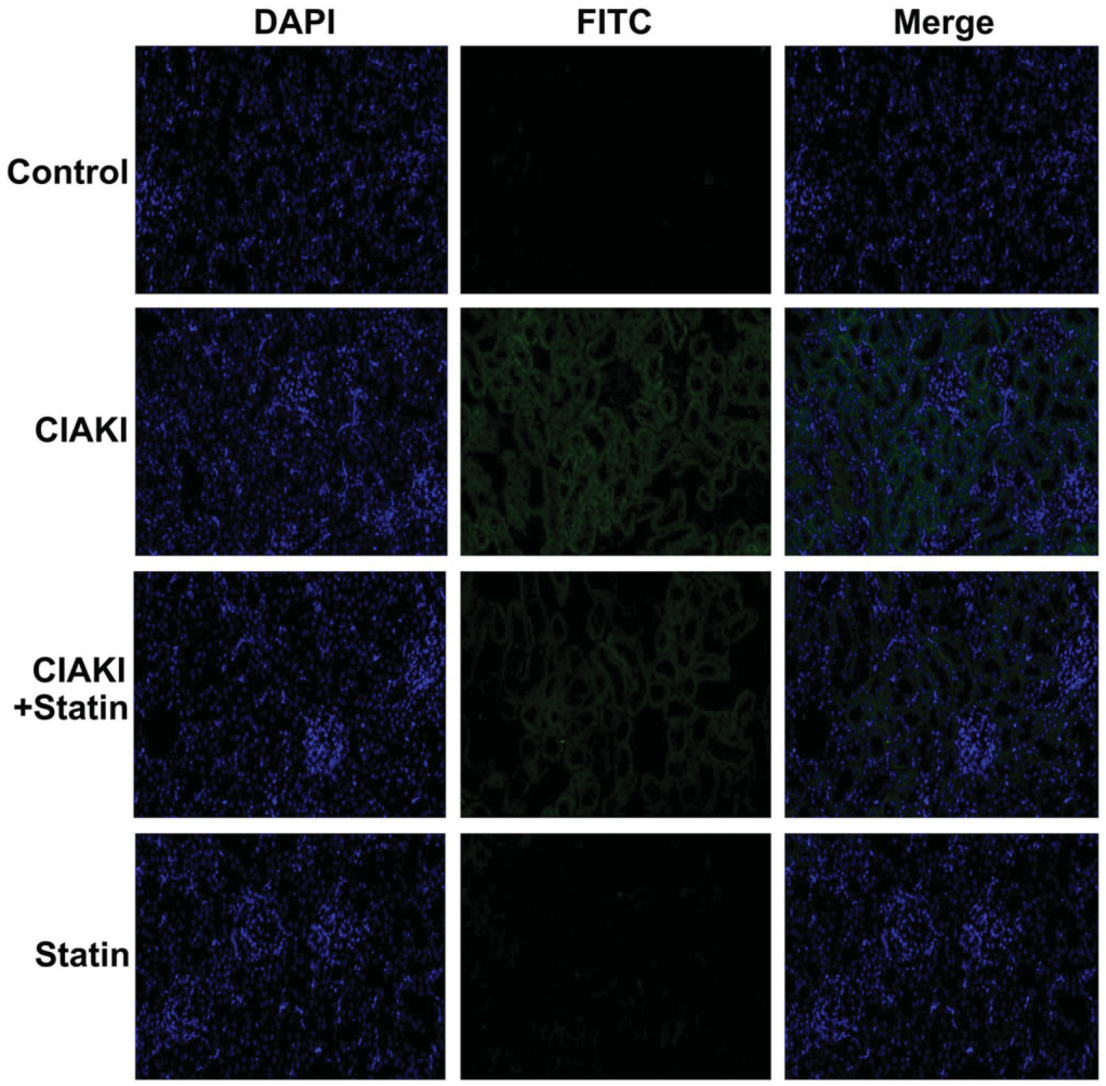
Atorvastatin protects against apoptosis in rat CIAKI model. TUNEL staining was used to investigate tubular apoptosis. Iopromide increased the number of TUNEL-positive renal tubular cells at 48 h after CM administration. Rats with atorvastatin pretreatment presented significantly less TUNEL-positive renal tubular cells.

**Figure 5.**
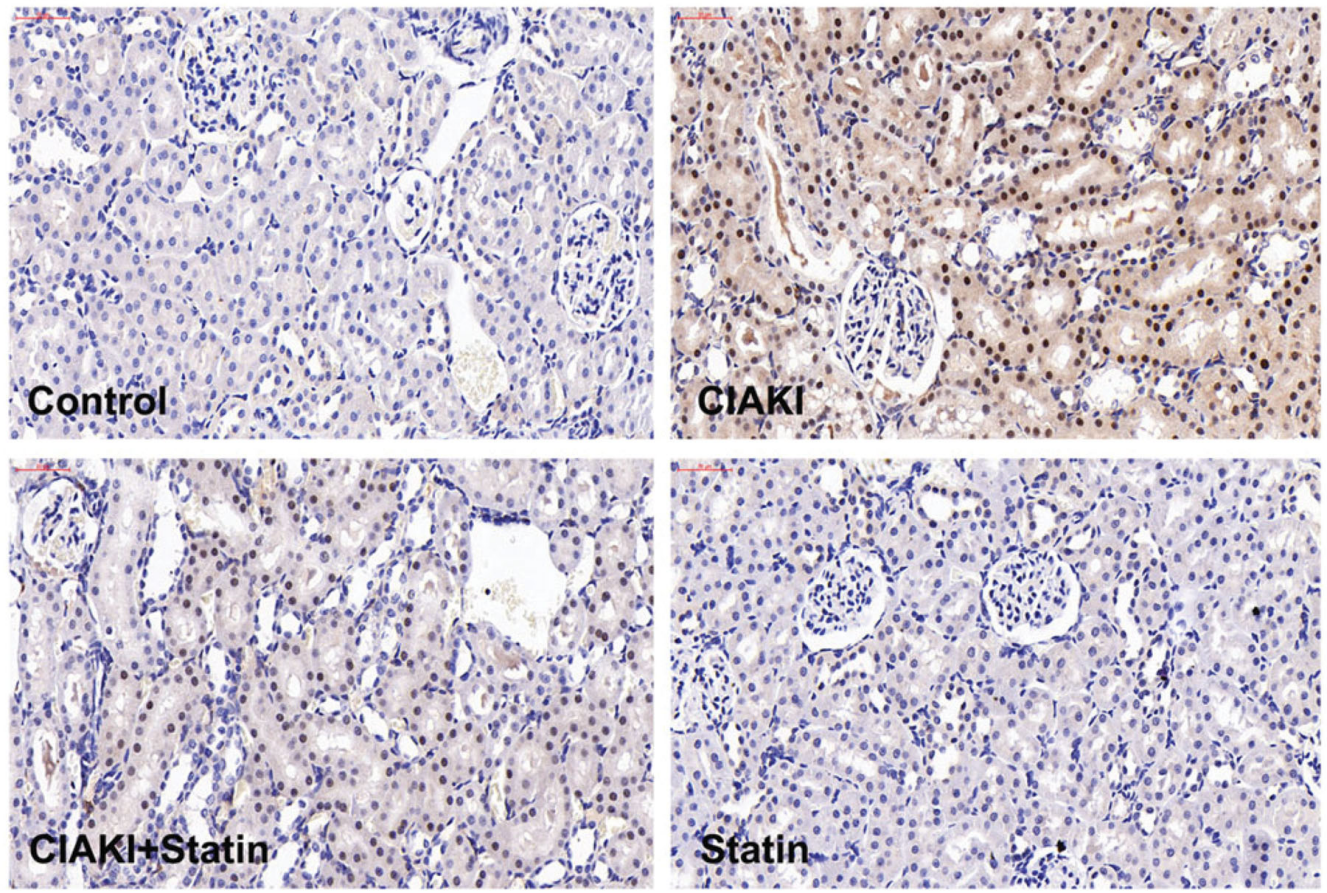
Atorvastatin dampened contrast-induced Caspase-3 expression in rat CIAKI model. Expression of Caspase-3 was demonstrated by immunohistochemical staining. In CIAKI group, expression of Caspase-3 was enhanced in renal tubular region. But in CIAKI + statin group, atorvastatin pretreatment dampened the contrast-induced Caspase-3 elevation.

CM also caused significant apoptosis, shown as increase in the number of TUNEL-positive tubular cells (Figure 4). Moreover, expression of Caspase-3 was simultaneously elevated in CIAKI rat kidney, which was detected by immunoblotting (Figure 5). Nevertheless, atorvastatin could profoundly alleviate CM-induced renal apoptosis, presenting as decreased number of TUNEL-positive tubular cells (Figure 4) and reduced expression of Caspase-3 (Figure 5). Atorvastatin alone had no effect on the number of TUNEL-positive tubular cells and Caspase-3 expression.

### Atorvastatin protects against inflammation in rat CIAKI model

**Figure 6.**
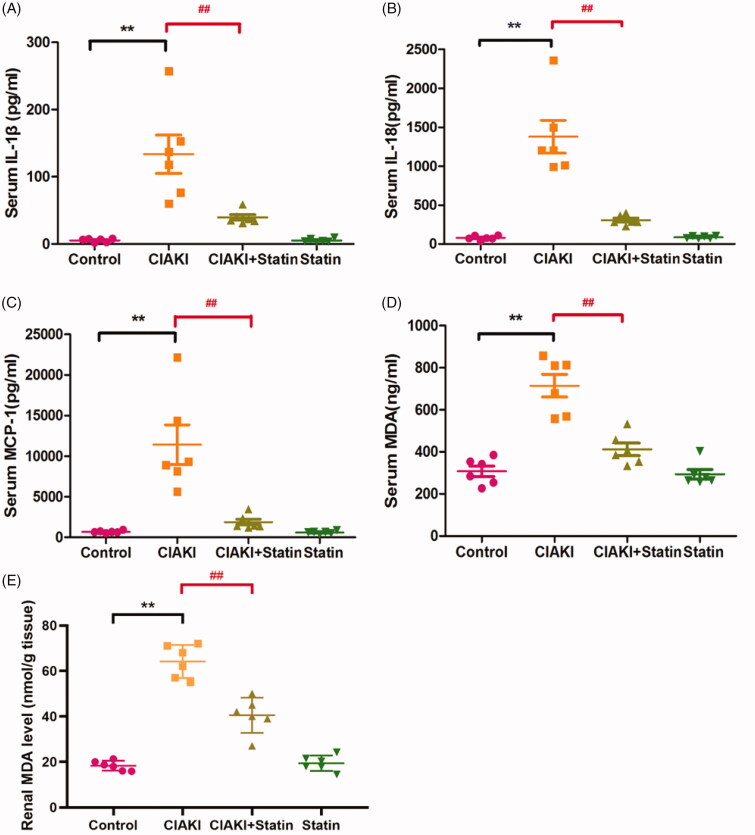
Atorvastatin protects against inflammation and oxidative stress in rat CIAKI model. Contrast caused systemic inflammation in rats, shown as elevated serum levels of IL-1β (A), IL-18 (B) and MCP-1 (C). Moreover, there was also higher level of MDA in CIAKI group both in serum (D) and kidney (E), representing excessive oxidative stress. Nevertheless, atorvastatin could attenuate contrast-induced expression of IL-1β, IL-18, MCP-1 and MDA. Data are presented as mean ± SD (n = 6). **p<0.01 versus control group. ##*p* < 0.01 versus CIAKI group.

In rat CIAKI model, systemic inflammation was significantly enhanced, shown as increase of several pro-inflammatory cytokines in serum, such as IL-1β, IL-18, and MCP-1 (Figure 6, *p* < 0.01). However, rats with atorvastatin pretreatment showed reduced expression of these three cytokines (Figure 6, *p* < 0.01), representing ameliorated inflammation. Atorvastatin alone had no effect on expressions of IL-1β, IL-18, and MCP-1.

### Atorvastatin protects against excessive oxidative stress in rat CIAKI model

Increase of MDA can induce oxidative reaction, and further induce cellular injury. In CM-treated rats, expressions of serum and renal MDA were both enhanced (Figure 6(D,E), *p* < 0.01), which represented excessive oxidative stress. At the same time, we also found a lower level of MDA in atorvastatin pretreated group, compared with CIAKI group (*p* < 0.01), showing the effectiveness of atorvastatin in reducing CM-induced oxidative stress. Atorvastatin alone had no effect on expressions of MDA.

### Atorvastatin increases H_2_S expression in rat CIAKI model

**Figure 7.**
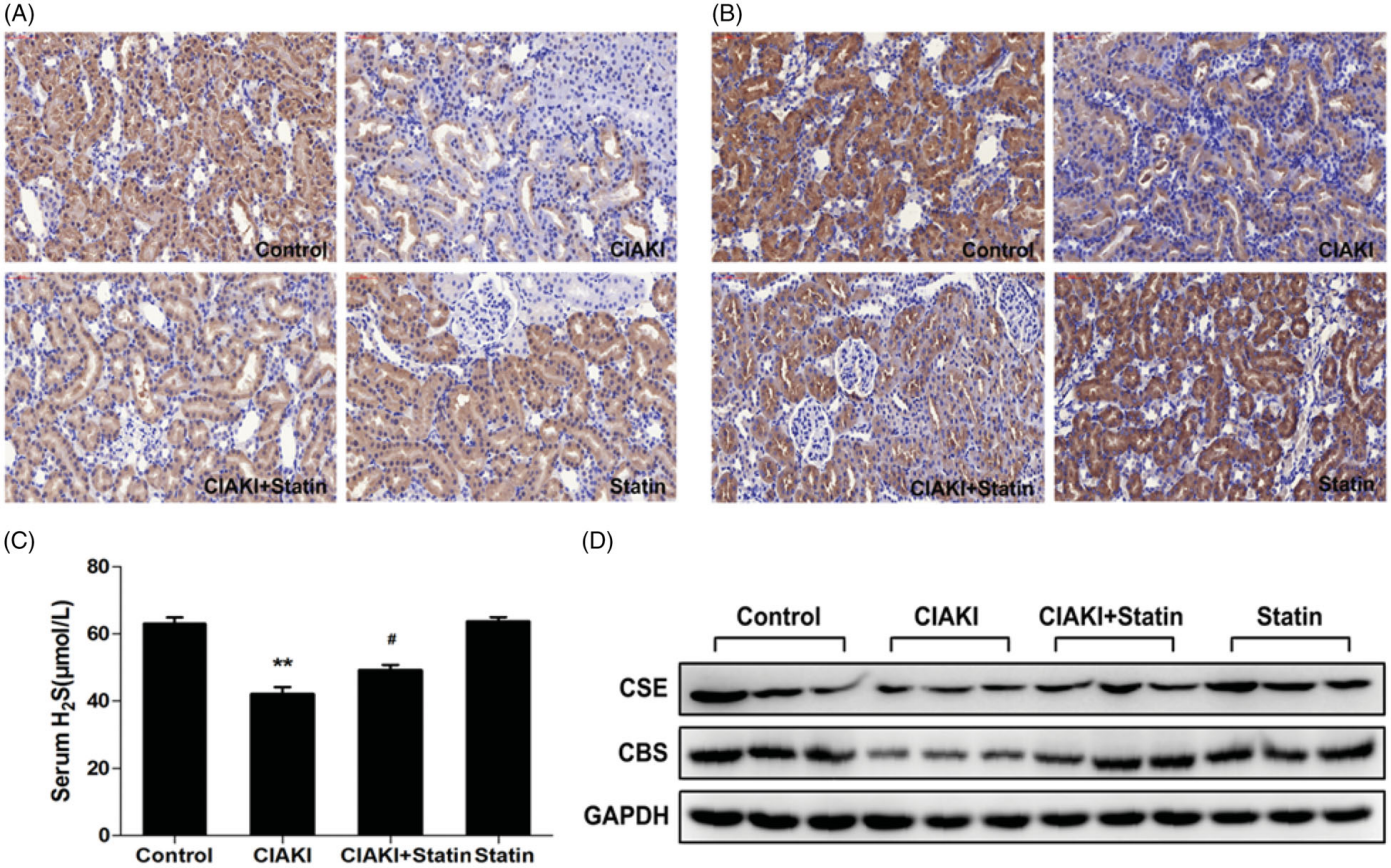
Atorvastatin restores H_2_S generation in rat CIAKI model. Serum H_2_S level was detected by ELISA kit (C). Two key enzymes of H_2_S generation in kidney, CSE and CBS, were quantified by Western blot analysis (D) and immunohistochemical staining (A for CSE, B for CBS). As shown, rats in CIAKI group had considerably lower serum H_2_S level, as well as deterioration of renal CSE and CBS expression. Atorvastatin could restore the systemic H_2_S expression and renal expression of CSE and CBS. Data are presented as mean ± SD (*n* = 6). **p < 0.01 versus control group. # *p* < 0.05 versus CIAKI group.

Furthermore, we investigated the systemic H_2_S level in CM-treated rats. We observed a remarkable decrease of serum H_2_S level in CIAKI group (Figure 7(C), *p* < 0.01). Interestingly, rats with atorvastatin pretreatment showed a restoration of H_2_S expression after CM injection (Figure 7(C), *p* < 0.05). Atorvastatin alone had no effect on H_2_S level.

### Atorvastatin increased CSE and CBS expression in rat CIAKI model

CSE and CBS are both expressed in rat kidney, which are the key enzymes involved in the production of endogenous H_2_S in kidney. In CM-treated rats, expressions of CSE and CBS were both suppressed in kidney, shown by western blot analysis (Figure 7(D)). Furthermore, immunohistochemical staining revealed that decrease of CSE and CBS expression were most prominent in proximal tubular cells (Figure 7(A,B)). However, atorvastatin pretreatment induced a profound rebound of CSE and CBS expression (Figure 7(A,B,D)), revealing that atorvastatin could restore tissue concentration of H_2_S in in CM-treated rat kidney. Atorvastatin alone had no effect on expressions of CSE and CBS.

### H_2_S protects against cell death in CM-treated NRK-52E cells

**Figure 8.**
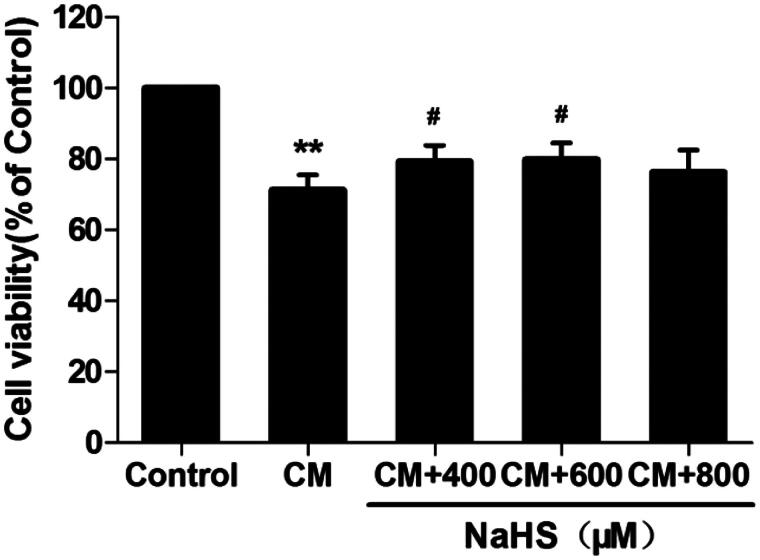
NaHS protects against CM-induced NRK-52E cell death. NRK-52E cells with CM showed significant cell death, presenting as decrease of cell viability. 400 and 600 μM NaHS pretreatment for 0.5 h could reverse the CM-induced cell death. Cell viability was assessed by CCK-8 analysis. Data are presented as mean ± SD (*n* = 3). **p < 0.01 versus control group. #*p* < 0.05 versus CM group.

According to our previous study [26], rat tubular epithelial cells (NRK-52E cells) were processed with 150 mg I/ml iopromide for 3 h to establish *in vitro* CM injury model. We observed decreased cell viability (by CCK-8 analysis, Figure 8, *p* < 0.01) in CM-treated NRK-52E cells. In order to further explore the mechanism of H_2_S pathway on CIAKI model, we tested the effect of NaHS (a donor of H_2_S) with different concentrations on CM-treated NRK-52E cells. Amazingly, pretreatment with NaHS from 400 to 600 μM significantly attenuated CM-induced cell death, shown as better cell viability (*p* < 0.05). H_2_S alone had no effect on cell viability in NRK-52E cells.

### H_2_S protects against inflammation in CM-treated NRK-52E cells

**Figure 9.**
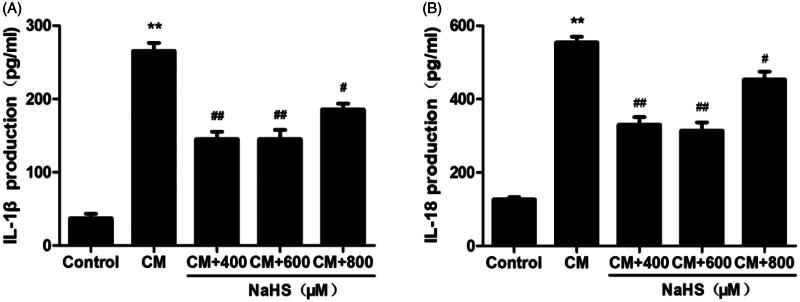
NaHS protects against CM-induced inflammation in NRK-52E cells. CM caused secretion of proinflammatory cytokines IL-1β and IL-18. However, they were attenuated by 400 μM and 600 μM NaHS pretreatment for 0.5 h. Supernatant IL-1β and IL-18 levels were detected by ELISA analysis. Data are presented as mean ± SD (*n* = 3). **p < 0.01 versus control group. #*p* < 0.05 versus CM group. ##*p* < 0.01 versus CM group.

As the systemic inflammation was stimulated in rat CIAKI model, we therefore investigated the localized inflammation in CM-treated tubular cells. As presented in Figure 9, the levels of IL-1β and IL-18 in supernatant were simultaneously elevated after CM treatment (*p* < 0.01). Meanwhile, 400 to 800 μM exogenous H_2_S dampened localized inflammation, presenting as decreased levels of IL-1β and IL-18 (*p* < 0.05 to *p* < 0.01).

## Discussion

In this study, we investigated the mechanism of atorvastatin protecting against CIAKI both *in vivo* and *in vitro*, and we found atorvastatin ameliorated the CM-induced injury of rat renal function and morphology, as well as stimulation of apoptosis, excessive oxidative stress and inflammation. Moreover, atorvastatin could improve the systemic level of H_2_S and localized expression of CSE and CBS, which were all dampened by CM. At last, *in vitro* study showed in rat tubular epithelium, H_2_S could protect against CM-induced cell death and inflammation. All these collecting results lead to our conclusion that atorvastatin protects against contrast-induced acute kidney injury *via* upregulation of endogenous hydrogen sulfide.

Among the various drug-induced acute kidney injuries in hospital, CIAKI is of the most frequent ones [27,28]. Although the mechanisms of CIAKI remains poorly understood, a series of studies has shown a toxic effect of CM on renal tubule [26,28–31]. CM can be taken up into the cells and damage mitochondrial function resulting in the increased generation of reactive oxygen species, release of proinflammatory cytokines and therefore cell apoptosis [27]. In our study, we also showed profound stimulation of oxidative damage and inflammation, which further induced cell apoptosis and renal dysfunction in CIAKI *in vivo* and *in vitro* models. CM destroys renal function and hence causes pathological damage including necrosis of renal tubular epithelial cells, proteinaceous casts in renal tubules, and medullary congestion [32], which are in line with our findings.

Atorvastatin was already shown to be effective in preventing CIAKI in patients undergoing coronary intervention [11]. Previous studies [33,34] have revealed that atorvastatin protects against CIAKI *via* inhibition of renal tubular apoptosis, which might be regulated by Hsp27, Akt, and ERK pathway. In our study, we got a similar observation (Figure 4). Beyond this, our results also demonstrated that CM induced an increase in oxidative stress. In many cellular systems [35,36], atorvastatin was reported to reduce the intracellular oxidative stress by acting on the inhibition of ROS producing enzymes. In this aspect, atorvastatin might protect against CIAKI by scavenging ROS. Furthermore, one of the pleiotropic effect of statin is decreasing synthesis of proinflammatory cytokines and chemokines [37]. In *in vivo* CIAKI model, we also observed decreased expression of proinflammatory cytokines, such as MCP-1, IL-1β, and IL-18, in rats with atorvastatin pretreatment. Therefore, anti-inflammation may be another crucial role in atorvastatin’s protection against CIAKI.

In kidney, H_2_S is produced through four pathways, while CSE and CBS are the two dominated enzymes for its generation [38]. In physiological conditions, H_2_S was found to inhibit sodium transporters on renal tubular cells, and thus regulate the excretory function of the kidney [39]. Likewise, it also influences the release of renin from juxtaglomerular cells and thereby modulates blood pressure [40]. What’s more, H_2_S modulates urine concentration by upregulating renal AQP-2 protein expression [41]. Liu et al. [42] recently showed that both CSE and CBS levels were severely decreased in a cisplatin-treated rats. Moreover, in renal ischemia/reperfusion models, supplement of H_2_S exerted protective effect likely through anti-inflammatory, anti-apoptotic, and anti-oxidative responses [43,44]. In our study, serum level of H_2_S, as well as renal expression of CSE and CBS, were significantly reduced in the CM-treated rats, paralleling to the renal injury. This indicated a decrease of systemic and localized generation of H_2_S in CIAKI model. Furthermore, when a H_2_S donor, NaHS, was employed in NRK-52E cells, CM-induced apoptosis and inflammation were both ameliorated remarkably. Therefore, our data prompted that H_2_S may also exhibited protection against CIAKI by anti-inflammatory, antiapoptotic, and antioxidative mechanism.

Interestingly, we also observed that atorvastatin could upregulate H_2_S expression in rat CIAKI models, as the systemic level of H_2_S and renal expression of CSE and CBS were simultaneously elevated with atorvastatin pretreatment. Actually, previous studies have shown that atorvastatin increases tissue concentration of H_2_S, such as kidney [18], liver [18], periaortic adipose tissue [45], and macrophages [37]. The mechanism of statin-induced increase in H_2_S might include stimulation of H_2_S synthesis by Akt signaling pathway [37] and inhibition of mitochondrial H_2_S oxidation by reducing CoQ [46,47]. What’s more, in raw264.7 macrophages, Xu et al. [37] revealed that suppression of CSE–H_2_S pathway abolished the anti-inflammatory action of fluvastatin, indicating that H_2_S system may serve as another important target contributing to the anti-inflammatory action of statins. Likewise, the atorvastatin-induced upregulation of H_2_S expression was paralleling to protection of renal function and morphology, as well as inhibition of inflammation and oxidative stress in our study. All these collective results suggested that in CIAKI, atorvastatin exerted its protective effect *via* upregulation of endogenous H_2_S.

## Conclusion

Our study investigated the mechanism about how atorvastatin protected against CIAKI and tried to figure out the possible role of endogenous H_2_S in its protection. Finally, we presented a hitherto unreported finding that atorvastatin protected against CIAKI *via* upregulation of endogenous H_2_S, both *in vivo* and *in vitro*.
